# Supplementary material for: Temporal and spatial distributions and clustering features of soil-transmitted helminthiases on Hainan Island: A retrospective study from 2017–2023
Source: PLoS Negl Trop Dis. 2026 Jan 7;20(1):e0013864. doi: 10.1371/journal.pntd.0013864 (PMC12810906; doi:10.1371/journal.pntd.0013864)
Supplement: Attachment — Submitted filename: Response to Reviewers.docx [file pntd.0013864.s004.docx]

October 30, 2025

Editorial Office

PLOS Neglected Tropical Diseases

Subject: Revision Response for Manuscript

ID: PNTD-D-25-01212

Title: Temporal and spatial distributions and clustering features of soil-transmitted helminthiases on Hainan Island: a retrospective study from 2017–2023

Dear Editors and Reviewers,

First and foremost, we would like to express our sincere gratitude to you and the reviewers for your valuable time, constructive comments, and professional suggestions on our manuscript (Manuscript ID: PNTD-D-25-01212; Title: Temporal and spatial distributions and clustering features of soil-transmitted helminthiases on Hainan Island: a retrospective study from 2017–2023). These comments have been of great significance for improving the quality and rigor of our work. We have carefully studied each comment and made corresponding revisions to address all the concerns raised. Below is a detailed response to each comment from the reviewers.

**Response to Editor**

Thank you for your reminder. In accordance with the requirements of *PLOS Neglected Tropical Diseases*, we have added an Author Summary section and ensured that the CRediT author contribution information for each co-author is accurate and complete. For the maps: All our figures are in .tif format and have passed testing with the NAAS tool. Additionally, our maps have successfully obtained the review number from the Hainan Provincial Bureau of Geomatics and Surveying: 琼S (2025) 249, which resolves all potential map copyright issues.Meanwhile, we have reconfirmed the consistency of funder and grant number details between the Financial Disclosure section and the Funding Information tab in the submission form, and clarified the extent of funders’ involvement in the study.

**Response to Reviewer 1**

**1. Reviewer Comment 1** : The study, "Temporal and spatial distributions and clustering features of soil-transmitted helminthiases on Hainan Island: a retrospective study from 2017–2023", is highly relevant for the epidemiological survey of major helminthiases in a tourist region with a climate favorable to the spread of these neglected parasites.

However, I noticed the absence of information regarding the time of year in which the stool samples were collected—whether during the rainy or predominantly sunny season—and whether participants had recently traveled to another region.

In conclusion, this is an excellent study that underscores the importance of monitoring neglected helminthiases and of implementing effective transmission control measures.

**Our Revision :** Thank you for your suggestion. Since the sampling process was continuous, the raw data included sample collection times. Most samples were collected during the rainy season (24,513 / 29,669). In Hainan, the rainy season spans from May to November, while the dry season runs from December to April of the following year. During sample collection, we primarily included individuals who had resided locally for at least 6 months, had not taken anthelmintic drugs, and were not experiencing diarrhea. Information on participant selection criteria has been added to the Methods section. Specific sample collection times, as well as basic information on the rainy and dry seasons, have been supplemented in the Results section. Additionally, the Discussion has been updated with relevant content addressing seasonal factors. More detailed information is available in Supplementary File 1.

**Response to Reviewer 2**

**1. Reviewer Comment 1 :** Sampling design and representativeness

- Need explicit site/village selection methods, randomisation/stratification, age distribution (especially 3–10 years), response rates, and any weighting.

**Our Revision :** Thank you for your suggestion. For the selection of survey sites/villages, we used a stratified random sampling approach. Each survey site/village was required to enroll no fewer than 200 participants. The specific procedures were as follows: All townships within each county were first categorized into five geographic regions (east, west, south, north, and central). From each geographic region, one township was randomly selected as a survey township. Finally, one administrative village was randomly selected from all administrative villages in the chosen township.

The overall inclusion criteria remained consistent with our previous revision: we primarily included individuals who had resided locally for at least 6 months, had not taken anthelmintic drugs, and were not experiencing diarrhea. There were no a priorispecific requirements for the age distribution of children aged 3–10 years during the survey. The response rate met the standards of the National Survey on Human Parasitic Diseases(≥85%). Currently, no weight matching was applied to the infection rate data.

These revisions have been updated in the Materials and Methods section.

**2. Reviewer Comment 2 :** Diagnostic methodology and case ascertainment

- Specify stool sample count per participant, additional diagnostics, test sensitivity/specificity, infection intensity data, and potential measurement biases.

**Our Revision :** Thank you for your suggestion. Each participant provided no less than 30 g of stool. Each sample was tested using the modified Kato-Katz technique, with a double slide examination per stool sample. All examiners were experienced and qualified microscopy experts: for each sample, one expert examined and counted one slide, while another expert examined and counted a second slide. Finally, data statisticians performed the statistical analysis.

After calculating eggs per gram (EPG), we found that: 96.64% of hookworm infections were light-intensity (EPG < 2000); 74.44% of *Ascaris lumbricoides* infections were light-intensity (EPG < 5000); and 92.03% of *Trichuris trichiura* infections were light-intensity (EPG < 1000). Only the infection rate was calculated for *Enterobius vermicularis*.

We did not measure the test sensitivity or specificity of each examiner initially; instead, examiners were required to undergo repeated training until they achieved: (1) 90% accuracy in identifying negative/positive samples, and (2) 100% recognition of *Ascaris lumbricoides*, *Trichuris trichiura*, hookworm, and clonorchiasis eggs to be deemed qualified.

Diagnostic criteria: A slide was classified as positive if at least one type of helminth egg was detected (mixed infections were possible), and infection rates were reported by species.

Potential measurement biases include: (1) missed detections in samples with low infection intensities, and (2) the Kato-Katz technique’s susceptibility to underdetection and difficulty in re-examination for hookworm eggs, which may lead to potentially underestimated infection rates.

This information has been added to the Materials and Methods and Discussion sections, respectively.

**3. Reviewer Comment 3 :** Temporal trends and confounders

- Discuss time-varying confounders (sanitation, deworming programs, economic changes, climate) and, if possible, analyze correlations with these factors.

**Our Revision :** Thank you for your suggestion. We have added relevant content to the Discussion section. However, we have not yet collected comparable information on time-varying confoundersm, so we cannot conduct an in-depth analysis of their relationship with the decline in infection rates.

According to previous relevant studies, economic growth and poverty alleviation efforts have significantly contributed to reducing infection rates by improving rural sanitation conditions. Preliminary investigations reveal that the coverage rate of sanitary toilets in rural Hainan has increased from 86.3% in 2016 to 99.8% in 2023, with a parallel rise in tap water access and substantial improvements in living environments. As a result, many areas that previously had high infection rates have seen a gradual decrease over time.

**4. Reviewer Comment 4 :** Spatial analysis details

- Provide more methodological specifics: scale/distance thresholds, handling of spatial autocorrelation, validation via simulations, interpretation of LISA clusters, and avoid ecological fallacy.

**Our Revision :** Thank you for your suggestion. We have provided similar parameters in the Materials and Methods section and Supplementary File 2.

**5. Reviewer Comment 5 :** Demography interpretation

- Offer hypotheses for sex/ethnicity/age patterns, consider socioeconomic confounding, and include age-specific or intensity analyses if available.

**Our Revision :** Thank you for your suggestion. We have provided a detailed discussion on population distribution in the manuscript. However, age-stratified analysis or analysis related to exposure intensity or disease severity is not feasible for this study.

**6. Reviewer Comment 6 :** Significant changes in prevalence and demographic factors.

Explain the causes of these changes

**Our Revision :** Thank you for your suggestion. We have added relevant content to the Discussion section to explain these changes. The observed shifts in prevalence and demographic factors are attributed to not only the full coverage of sanitation infrastructure projects but also the increased availability of more effective anthelmintic drugs and strengthened health education campaigns that have promoted improved hygiene practices.

**7. Reviewer Comment 7 :** Policy implications

- Add concrete, actionable recommendations (targeted school-based deworming, sanitation improvements, health education) and a monitoring framework with interim targets.

**Our Revision :** Thank you for your suggestion. We have added implementation plans to the Discussion section. To address the high *Enterobius vermicularis* infection rates in children, we propose collaborating with other public health programs targeting children to conduct regular free pinworm testing for those aged 3–10. For infected children, we suggest providing targeted free anthelmintic medication—or integrating post-enrollment hand washing with smart toilet anal cleansing—to reinforce prevention. Additionally, agricultural workers, women, and individuals aged >60 years represent a highly overlapping high-risk group. We recommend that the health department collaborate with the rural agriculture department to launch health education campaigns and anthelmintic treatment initiatives, while integrating fecal examination services into basic public health programs.

**8. Reviewer Comment 8 :** Limitations and biases

- Expand to seasonal effects, diagnostic variability, migrant populations, data handling, and propose robust sensitivity analyses.

**Our Revision :** Thank you for your suggestions. We have expanded these points in the Discussionsection.

This study was primarily based on a cross-sectional survey, which may have introduced some selection bias. Most samples were collected between February and November. The rainy season on Hainan Island spans May–November, while the dry season runs from December to April of the following year. Rainy-season samples accounted for 82.62% of all collections. However, the positivity rate of dry-season samples was higherthan that of rainy-season samples—meaning infection rates may have been underestimated to some extent.

Additionally, hookworm is the predominant STH in Hainan. Due to the biological characteristics of hookworm eggs, their detection sensitivity using the modified Kato-Katz techniqueis relatively lower than that of *Ascaris lumbricoides* or *Trichuris trichiura* eggs. Manual microscopy also has inherent variability in detection capability, which may have led to actual test results being lower than the true infection rate.

Furthermore, spatial statistical methods (e.g., spatial autocorrelation analysis) used here require global datato reliably explain regional distribution patterns of STH prevalence. Applying these methods to sampled data may introduce bias. Spatial interpolation techniques could also average out local outliers, masking high-risk areas at small spatial scales.

This study included only residents who had lived locally for ≥6 months; migrant populations were excluded during sampling. We will add this clarification to the Methodssection. To improve detection reliability, future studies could use more sensitive tools (e.g., rapid diagnostic test strips or automated fecal analysis machines) to reduce variability in manual testing.

In summary, these factors may explain discrepancies between our results and the true STH infection status in Hainan. Nevertheless, our study still captures key features of STH transmission in the region.

**9. Reviewer Comment 9 :** Reproducibility and transparency

- Provide data dictionary, data access plan, and share code or software details and parameters used.

**Our Revision :** Thank you for your suggestion. We have submitted our raw data as Supplementary File 1. Details of software parameters can be included in Supporting Information File 2.

**Response to Reviewer 3**

**1. Reviewer Comment 1 :** The study states that, according to the national surveillance protocol, “two mobile and one fixed sites” were selected to cover 18 counties/cities. However, is the balance between mountainous vs. coastal areas and urban vs. rural settings adequately ensured?

**Our Revision :** Thank you for your question. Based on the geographical distribution of Hainan Island, we conducted surveys across different monitoring sites each year—the overall data provide reasonable assurance of balance between mountainous/coastal areas and rural/urban settings. Additionally, each monitoring site includes 5 survey villages/sites, and each survey site employs a five-directional sampling strategy(east, south, west, north, and center) to ensure balanced coverage within each county. Together, these two measures enhance the representativeness and balance of our sample.

**2. Reviewer Comment 2 :** The Kato–Katz thick smear method has limited sensitivity for light infections, especially in detecting hookworm and *Ascaris lumbricoides*, which may lead to underestimation of prevalence. This study did not incorporate molecular methods (e.g., qPCR) or multiple sampling for validation. The reported Kato–Katz detection rate was only 0.38%, whereas the perianal tape method in children yielded 6.47%—a striking discrepancy.

**Our Revision :** Thank you for your comment. Molecular qPCR methods certainly have higher sensitivity than the Kato–Katz thick smear technique; however, given the large sample size of this surveillance study and cost considerations, the thick smear method was more appropriate. For future monitoring studies, we could select a subset of samples for re-testing to validate our findings, which would complement the current experimental design.Additionally, *Enterobius vermicularis*primarily lays eggs around the anus, so the perianal tape test indeed has a higher detection rate than fecal examination.

**3. Reviewer Comment 3 :** Annual infection rates showed a “rebound” in 2020 (from 4.56% in 2019 to 8.72%), yet the authors concluded an overall decline based solely on χ² trend tests. Additional sensitivity analyses or more robust models such as Joinpoint regression or Poisson regression should be applied.

**Our Revision :** Thank you for your suggestion. We have revised our analysis to use Joinpoint regression to examine the temporal trend of annual infection rates. The results show that both STH (AAPC = -18.99, P < 0.05) and hookworm (AAPC = -32.61, P < 0.05) exhibit a statistically significant declining trend, while trends for other species are not statistically significant (all P > 0.05).

**4. Reviewer Comment 4 :** Global Moran’s I was significant only for *Trichuris trichiura* (I = 0.137, P = 0.047), whereas local spatial clustering identified high–high/low–low clusters for multiple species. Could the lack of significance at the global level be due to low overall prevalence and limited statistical power? The study should clarify the choice of spatial weight matrices and apply significance correction (e.g., FDR) to avoid potential false positives.

**Our Revision :** Thank you for your insightful comments. The primary factor contributing to the non-significant global Moran’s I is the low overall prevalence of STH. Low prevalence attenuates the global spatial association of species distributions, making it challenging for the global Moran’s I to detect significant signals. However, local areas may still exhibit significant clustering (e.g., high-high/low-low clusters) due to higher aggregation at smaller spatial scales.Regarding the spatial weight matrix, we set it to INVERSE_DISTANCE. We will include the software parameter details in Supporting Information File 2. Regarding significance correction, we will apply the False Discovery Rate (FDR) method to adjust for multiple comparisons in future analyses to reduce false positives.

**5. Reviewer Comment 5 :** The higher hookworm prevalence was attributed to “barefoot farming,” but the analysis lacks quantitative exploration of other ecological factors such as climate, soil moisture, and behavioral differences among migrant populations.

**Our Revision :** Thank you for raising this important point. This is a limitation of our study—we linked higher hookworm prevalence to 'barefoot farming' but did not quantitatively explore other ecological factors (e.g., climate, soil moisture) or migrant behavior. Given that climate and soil affect infection rates, we will use our surveillance findings to conduct follow-up analyses. Importantly, all participants were permanent residents (≥6 months), excluding short-term migrants (<6 months), which minimizes confounding from population mobility.

**6. Reviewer Comment 6 :** The study reports higher prevalence in Han compared to Li ethnic groups, contrary to historical surveys, yet attributes this difference solely to “improved sanitation in minority regions.” Is there supporting data for this explanation?

**Our Revision :** Thank you for your question. The higher prevalence in the Han vs. Li ethnic group—contrary to historical surveys—is not solely due to “improved sanitation in minority regions”; we only outlined potential contributors. The Li population is concentrated in south-central Hainan, where recent investments in sanitation infrastructure (e.g., sanitary toilets rising from 78.3% in 2016 to 99.8% in 2023, near-universal tap water access) have expanded. We’ve refined the text to clarify this context.

**7. Reviewer Comment 7 :** The authors note the national criterion (<1% prevalence sustained for three consecutive years to indicate transmission control), but prevalence remained >3% during 2021–2023. This suggests Hainan has not yet achieved transmission control. What specific public health strategies (e.g., targeted deworming of key populations, school-based health education, or environmental improvements) should be considered?

**Our Revision :** Thank you for your question. Consistent with our previous revision, we have added implementation plans to the Discussionsection: To address the high *Enterobius vermicularis* infection rates in children, we propose collaborating with other public health programs targeting children to conduct regular free pinworm testing for those aged 3–10. For infected children, we suggest providing targeted free anthelmintic medication—or integrating post-enrollment hand washing with smart toilet anal cleansing—to reinforce prevention. Additionally, agricultural workers, women, and individuals aged >60 years represent a highly overlapping high-risk group. We recommend that the health department collaborate with the rural agriculture department to launch health education campaigns and anthelmintic treatment initiatives, while integrating fecal examination services into basic public health programs.

**8. Reviewer Comment 8 :** The study mainly compares its findings with surveillance data from other Chinese provinces, but offers little comparison with international studies (e.g., tropical island countries in Southeast Asia). Such comparisons would strengthen global relevance.

**Our Revision :** Thank you for your suggestion. We have added comparisons with countries such as Malaysia, Thailand, the Philippines, and Ethiopia in the Discussionsection.

**9. Reviewer Comment 9 :** The KDE and LISA spatial clustering maps are informative, but the links between spatial patterns and local ecological/economic conditions are insufficiently discussed. For example, why did Wuzhishan emerge as a core high-density zone? Could this be associated with mountainous rainfall, soil pH, or farming practices?

**Our Revision :** Thank you for your suggestion. According to the annual statistical yearbook of Wuzhishan City, it is located in the south-central part of Hainan Island with a mountainous terrain. It has an annual average temperature of 23.7°C and annual precipitation of 1,483.1 mm, with rainfall coinciding with the warm season. The city primarily relies on a mountainous agricultural model, cultivating cash crops such as areca nuts and rubber, which require frequent field fertilization and thus greater soil contact—conditions more favorable for the proliferation of STH compared to other cities and counties. We have added relevant content to the Discussionsection to address this.

All revisions have been marked in red in the revised manuscript for your easy identification. We believe the revised version has significantly improved the clarity, completeness, and scientific validity of the study. We hope this revision meets the requirements of *PLOS Neglected Tropical Diseases*.

Should you or the reviewers have any further questions or need additional clarification, please do not hesitate to contact the corresponding author:

YuChun Li

Hainan Provincial Center for Disease Control and Prevention

Email : [yuchunlee@126.com](mailto:yuchunlee@126.com)

Sincerely,

YuChun Li

Corresponding Author
